# Adenine inhibits growth of hepatocellular carcinoma cells via AMPK-mediated S phase arrest and apoptotic cascade

**DOI:** 10.7150/ijms.42086

**Published:** 2020-02-24

**Authors:** Wei-Wen Su, Jen-Yu Huang, Han-Min Chen, Jiun-Tsai Lin, Shao-Hsuan Kao

**Affiliations:** 1 Division of Gastroenterology, Department of Internal Medicine, Changhua Christian Hospital, Changhua 50006, Taiwan.; 2 Institute of Biochemistry, Microbiology, and Immunology, Chung Shan Medical University, Taichung 40201, Taiwan.; 3 Institute of Applied Science and Engineering, Catholic Fu Jen University, New Taipei 24205, Taiwan.; 4 Energenesis Biomedical Co. Ltd., Taipei 11492, Taiwan.; 5 Institute of Medicine, Chung Shan, Medical University, Taichung 40201, Taiwan.; 6 Clinical Laboratory, Chung Shan Medical University Hospital, Taichung 40201, Taiwan.

**Keywords:** apoptosis, hepatocellular cell, adenine, AMPK, p53, p21, Bax

## Abstract

**Background:** Adenine exhibits potential anticancer activity against several types of malignancies. However, whether adenine has anticancer effects on hepatocellular carcinoma (HCC) cells is incompletely explored.

**Methods:** Human HCC cell lines HepG2 and SK-Hep-1 (p53-wild type) and Hep3B (p53-deficient) were used as cell model. Cell growth and cell cycle distribution were determined using MTT assay and flow cytometric analysis, respectively. Protein expression and phosphorylation were assessed by Western blot. Involvement of AMP-activated protein kinase (AMPK) was evaluated using specific inhibitor and small inhibitory RNA (siRNA).

**Results:** Adenine treatments (0.5 - 2 mM) clearly decreased the cell growth of Hep G2 and SK-Hep-1 cells to 72.5 ± 3.4% and 71.3 ± 4.6% of control, respectively. In parallel, adenine also induced sub-G1 and S phase accumulation in both HCC cells. However, adenine did not affect the cell growth and cell cycle distribution of Hep3B cell. Western blot analysis showed that adenine reduced expression of cyclin A/D1 and cyclin-dependent kinase (CDK)2 and upregulated p53, p21, Bax, PUMA, and NOXA in HepG2 cell. Moreover, adenine induced AMPK activation that was involved in the p53-associated apoptotic cascade in HepG2 cells. Inhibition of AMPK activation or knockdown of AMPK restored the decreased cell growth of HepG2 and SK-Hep-1 cells in response to adenine.

**Conclusions:** These findings reveal that adenine reduces the cell growth of HepG2 and SK-Hep-1 but not Hep3B cells, attributing to the AMPK/p53-mediated S phase arrest and apoptosis. It suggests that adenine has anticancer potential against p53-wild type HCC cells and may be beneficial as an adjuvant for HCC treatment.

## Introduction

Malignant cancers are life-threaten diseases that contribute to the major mortality of human. Among the various types of cancers, hepatocellular carcinoma (HCC) is the leading cause of death in the patients with liver cancer in Taiwan and in the world 1, 2. Although several targeted anticancer drugs and immunotherapy for HCC have been newly developed in the past decade, the overall survival of patients with HCC is not significantly extended. The high mortality rate caused by HCC may be attributed to the complicated combination of cirrhosis, thrombocytopenia, and neutropenia and a high frequency of chemoresistance, which greatly diminishes the therapeutic effect 3. Therefore, it still needs to develop novel therapeutic strategy with different mechanisms for the improvement of HCC treatment.

Previous studies have demonstrated that purine nucleotides are considered important signaling molecules involved in a wide range of physiological and biological activities 4, 5. For example, adenosine nucleoside plays a pivotal role in governing cell differentiation, cardiac function, and neurotransmission 6-8. In parallel, adenine, a purine nucleobase, has also shown various bioactivities through adenosine monophosphate-activated protein kinase (AMPK) signaling, including anti-inflammation and anti-cancer activities in our previous studies 9-11.

In light of the potential antitumor activity of adenine that has been reported, we further investigate the anticancer effects of adenine on HCC cells with emphasis on the possible underlying mechanisms. Cell viability and cell cycle distribution were determined using MTT assay and flow cytometric analysis, respectively. Signaling cascade was demonstrated by using Western blotting. Involvement of AMPK in anticancer potential of adenine was also assessed.

## Materials and Methods

### Chemicals, reagents, and antibodies

All chemicals, including adenine, dorsomorphin, 3-(4,5-Dimethylthiazol-2-yl)-2,5-diphenyltetrazolium bromide (MTT), and propidium iodide (PI) were obtained from Sigma-Aldrich (St. Louis, MO, USA). Cell culture reagents RPMI-1640 and fetal bovine serum (FBS) were obtained from Invitrogen (Carlsbad, CA, USA). Anti-human cyclin A (sc-271682), cyclin D1 (sc-20044), cyclin-dependent kinase (CDK)2 (sc-6248), p21 (sc-53870), Bax (sc-20067), p53 upregulated modulator of apoptosis (PUMA, sc-374223), β- actin (sc-8432), and peroxidae-conjugated secondary antibodies (sc-516102, sc-2357) were acquired from Santa Cruz Biotechnology (Santa Cruz CA, USA). Anti-human phospho(p)-AMPK(T172) (#50081), AMPK (#5832), p53 (#9282), and p-p53(S15)(#9284), p-p53(S46)(#2521) antibodies were purchased from Cell Signaling Technologies (Beverly, MA, USA).

### Cell culture and experimental treatments

The human HCC cell line HepG2 (HB-8065) and SK-HEP-1 (HTB-52) were acquired from American Type Culture Collection (ATCC; Rockville, MD) and maintained in accordance with recommendations in Dulbecco's modified Eagle's medium (DMEM) supplemented with 10% FBS. Cells reaching to 80% confluency were trypsinized and subcultured into 6-well plates at an initial density of 1x10^5^ cells/mL for the subsequent treatments.

Cells were incubated with serial concentrations of adenine (0.5, 1, and 2 mM) in serum-free DMEM for 24 h. After the incubation, the treated cells were washed with phosphate-buffered saline (PBS; 25 mM sodium phosphate, 150 mM NaCl, pH 7.2) and collected for following analyses. Three independent experiments were performed for statistical analysis.

### Cell viability assay

Cell viability was determined by MTT assay as previously described 12. After treating with adenine or the combination of adenine and specific inhibitor, the culture supernatant was aspirated and the cells were washed with PBS then incubated with MTT solution (0.5 mg/mL) at 37^o^C for 4 h. The number of viable cells was proportional to the production of formazan which was solubilized with isopropanol and assessed by determining the absorbance at 570 nm using a microplate reader (SpectraMAX 360 pc, Molecular Devices, Sunnyvale, CA, USA).

### Determination of cell cycle distribution

Cell cycle distribution was analyzed using flow cytometry. At the end of treatment, cells were washed with PBS, collected by centrifugation, and then fixed with 1 ml of ice-cold 70% ethanol. The fixed cells were incubated with the staining solution (20 μg/mL propidium iodide (PI), 20 μg/mL RNase A, and 1% Triton X-100) for 15 min in dark, and then the cells were analyzed using a FACS Calibur system (version 2.0, BD Biosciences, Franklin Lakes, NJ, USA) equipped with CellQuest software. Data from three independent experiments were used for statistical analysis.

### Western blot

After washed with PBS, cells were harvested and incubated with the RIPA buffer (20 mM Tris-HCl, pH 7.5, 150 mM NaCl, 1% Nonidet P-40, 1% sodium deoxycholate, 2.5 mM sodium pyrophosphate, 1 mM sodium vanadate, 1 μg/mL leupeptin, and 1mM Phenylmethanesulfonyl fluoride). After centrifugation at 20,000g for 20 min, the supernatant was collected and used as crude proteins. Protein concentration was determined using Bradford method (DC Protein Assay, Bio-Rad Laboratory, Hercules, CA, USA). Crude proteins (20 μg per lane) were separated by a 12.5% SDS-polyacrylamide gel and transferred onto a nitrocellulose membrane (Millipore, Bedford, MA). The membrane was blocked with 5% skimmed milk/PBS, incubated with primary antibodies (1000X-diluted), and then incubated with peroxidase-conjugated secondary antibodies. Signal development was conducted using ECL chemiluminescence reagent (Millipore) and the luminescent signals were acquired and semi-quantitated by an image analysis system (Fuji Film, Tokyo, Japan).

### AMPK silencing by small inhibitory RNA

AMPKα1 small interfering RNAs (siRNAs, 108454 and s100) and AMPKα2 siRNAs (50583 and s11058) were purchased from Life Technologies and used for AMPK knockdown. siRNAs were transfected into HepG2 and SK-Hep-1 cells in medium containing 10% FBS using Lipofectamine RNAiMax (Invitrogen) according to manufacturer's instructions, and then incubated at 37°C and 5 % CO_2_ for 72 h.

### Statistical analysis

Data were expressed as means ± standards deviations (S.D.). Statistical significance analysis was conducted by using One-way ANOVA followed by Dunnett for multiple comparisons with the control. The difference with *P* value less than 0.05 was considered as statistically significant.

## Results

### Effects of ENERGI-F706 on viability of HepG2, SK-Hep-1, and Hep3B cells

**Figure 1:**
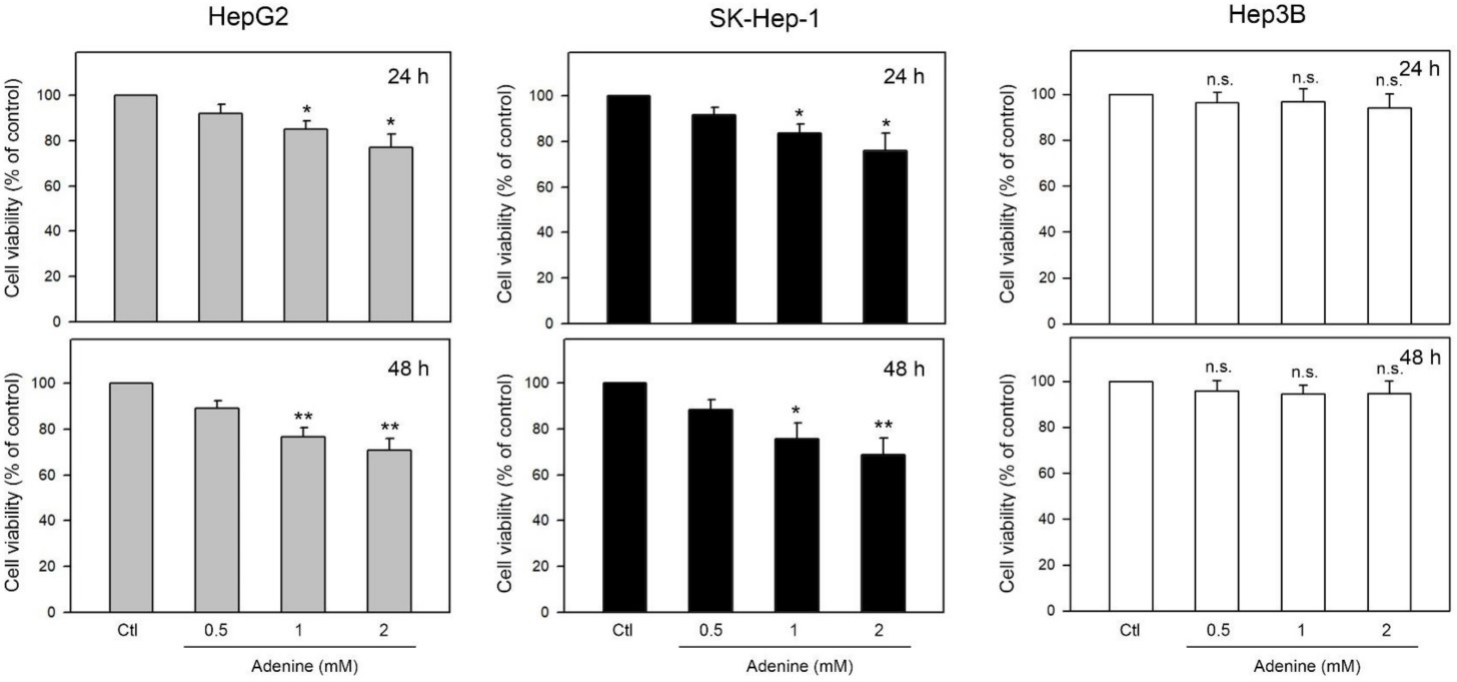
Effects of adenine on cell viability of HepG2, SK-Hep-1, and Hep3B cells. Cells were treated with adenine at serial concentrations (0.5, 1, and 2 mM) for 24 h (upper panel) or 48 h (lower panel), and then the cells were harvested for cell viability assay. Data were expressed as mean ± S.D. from three independent experiments. * and **, *P*<0.05 and *P* <0.01 as compared to the PBS control (Ctl).

Effects of adenine on cell viability of firstly determined by using MTT assay. Three human HCC cell lines including HepG2 and SK-Hep-1 (wild type p53) and Hep3B (p53 deficient) were tested. As shown in Figure 1, the cell viability of HepG2 and SK-Hep-1 cells were reduced up to 77.2 ± 6.7% and 75.7 ± 8.1% of control, respectively, by 24-h adenine treatments (*P* <0.05 as compared to control). In addition, 48-h adenine treatments further decreased the cell viability of HepG2 and SK-Hep-1 cells up to 71.6 ± 4.6% and 70.4 ± 7.9% of control, respectively (*P* <0.01 as compared to control). Interestingly, neither 24-h nor 48-h treatments of adenine significantly changed the cell viability of Hep3B cells (Figure 1, right panel). These observations show that the cell viability of HCC cells with wild type p53 is clearly reduced by adenine treatments in a dose-dependent manner.

### Adenine treatments resulted in cell cycle accumulation at sub-G1 and S phase in HepG2 and SK-Hep-1 but not Hep3B cells

**Figure 2:**
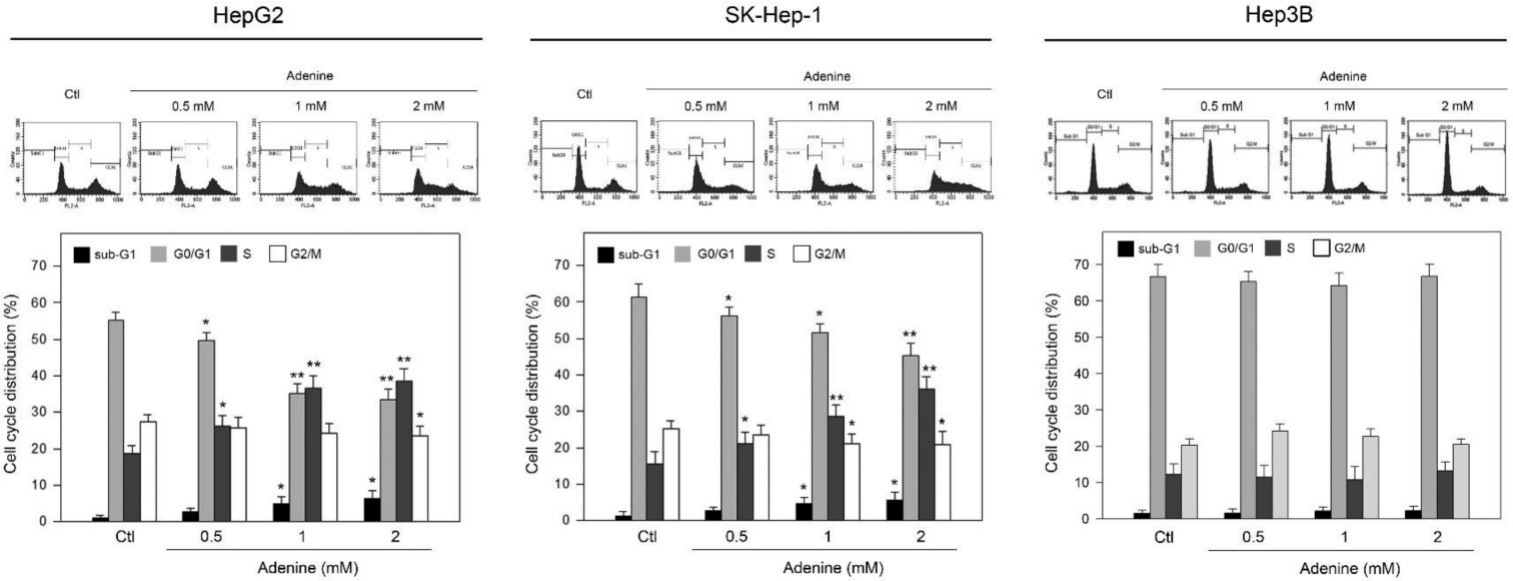
Effects of adenine on the cell cycle distribution of HepG2, SK-Hep-1, and Hep3B cells. Cells were treated with adenine at serial concentrations (0.5, 1, and 2 mM) for 24 h, and then the cells were harvested for flow cytometric analysis. Individual cell cycle phase was indicated. Data were expressed as mean ± S.D. from three independent experiments. * and **, *P*<0.05 and *P* <0.01 as compared to the PBS control (Ctl).

Since adenine reduced cell viability of HCC cells with wild type p53, the effects of adenine on cell cycle distribution were then explored. Our findings showed that adenine significantly increased the ratios of sub-G1 and S phases, while decreasing the ratio of G0/G1 phase (Figure 2). The average ratio of sub-G1 phase was increased to 3.3-fold and 3.1-fold in HepG2 and SK-Hep-1 cells in response to 2 mM adenine treatment, respectively (*P* <0.05 as compared to control). In parallel, the average ratio of S phase was increased to 2.1-fold and 2.5-fold in HepG2 and SK-Hep-1 cells, respectively, in response to 2 mM adenine treatment (*P* <0.01 as compared to control). Similar to the observation in cell viability, adenine did not influence the cell cycle distribution of Hep3B (Figure 2, right panel). Collectively, these findings reveal that adenine treatments induce the sub-G1 and S phase accumulation in the HCC cells with p53 but not in p53-deficient Hep3B cell.

### Adenine altered the expression of cell cycle regulators and induced apoptotic cascade in HepG2 and SK-Hep-1 cells

**Figure 3:**
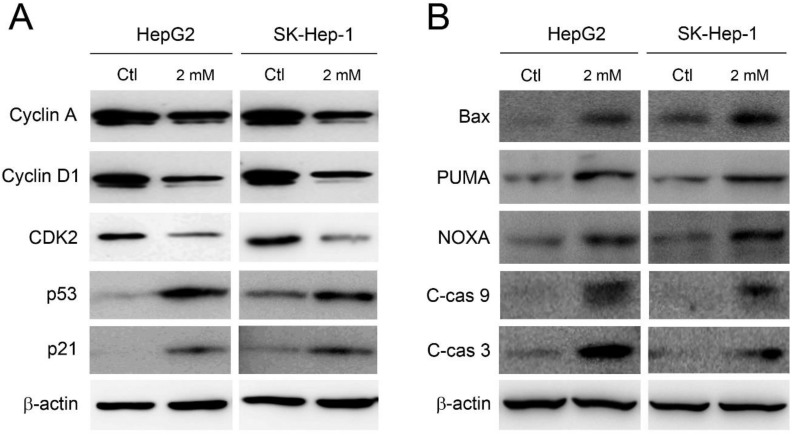
Adenine influenced the cell cycle regulators and induced apoptotic cascade in HepG2 and SK-Hep-1 cells. Cells were treated with 2 mM adenine for 24 h, and then the cells were used for Western blot analysis to determine the levels of proteins. PBS treatment was used as control (Ctl). C-cas 9, cleaved caspase 9 (37 kDa); C-cas 3, cleaved caspase 3 (19 kDa). β-actin was used as internal control.

The accumulation at sub-G1 and S phase implies the induction of apoptosis and the disruption of cell cycle. Therefore, the expression of cell cycle regulators and pro-apoptotic components was further investigated. As shown in Figure 3A, the expression level of cyclin A, cyclin D1, and CDK2 was clearly decreased in HepG2 and SK-Hep-1 cells by adenine treatments. Meanwhile, the expression of p53 and its downstream p21 were significantly increased in response to adenine treatment. In parallel, Bax, PUMA, and NOXA, which have been reported to be involved in the p53-mediated apoptosis 13, 14, were clearly upregulated in HepG2 and SK-Hep-1 cells in the presence of adenine (Figure 3B). Moreover, adenine also induced the cleavage of caspase 9 and caspase 3 (Figure 3B). Taken together, these results indicate that adenine alters the expression of cell cycle regulators which may further induce the p53-mediated cell cycle arrest and apoptotic cascade.

### Adenine induced activation of AMPK to trigger p53-mediated apoptotic signals in HepG2 cells

**Figure 4:**
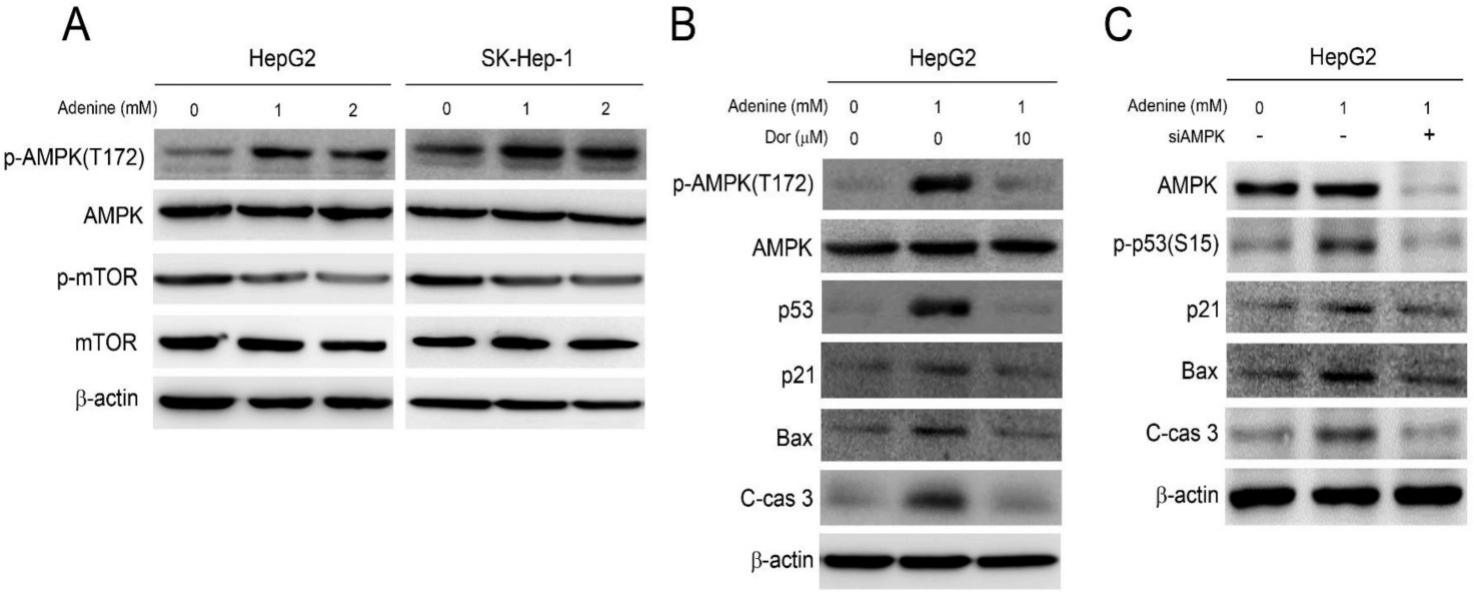
Adenine induced activation of AMPK and p53 involving in apoptotic cascade in HepG2 cells. (A) Cells were treated with 1 or 2 mM adenine for 24 h. (B) Cells were pre-treated with dorsomorphin for 2 h, and then treated with 1 mM adenine for 24 h. (C) Cells were transfected with scramble RNA or siAMPK, and then treated with 1 mM adenine for 24 h. The treated cells were collected and lysed for Western blot analysis. Dor, dorsomorphin; C-cas 3, cleaved caspase 3 (19 kDa). β-actin was used as internal control.

Our previous studies have demonstrated that adenine exerts its anticancer activity by activating AMPK and the associating signaling 10, 11. As a result, the role of AMPK activation in adenine-induced apoptotic signals in HepG2 cells was then investigated. As shown in Figure 4A, adenine treatment clearly resulted in phosphorylation of AMPK at threonine-172 (T172), and reduced the downstream mTOR phosphorylation. Next, the involvement of AMPK activation in adenine-induced cell cycle arrest and apoptotic signals was further examined. By pretreating with dorsomorphin (Dor), an AMPK inhibitor, the adenine-induced AMPK phosphorylation at T172 was significantly reduced in HepG2 cells (Figure 4B). In addition, dorsomorphin pretreatment also downregulated the expression level of p53, p21, and Bax and reduced the cleavage of caspase 3 in HepG2 cells with exposure of adenine (Figure 4B). We also silenced AMPK expression in HepG2 cell by using specific siRNA, and the results showed that the adenine-induced p53 phosphorylation at serine-15 (S15), p21, Bax, and cleaved caspase 3 were diminished in response to AMPK knockdown (Figure 4C). Collectively, these findings reveal that adenine-induced AMPK activation is involved in p53/p21/Bax-associated apoptotic signals in HepG2 cells.

### Adenine reduced the cell viability and induced S phase arrest in HepG2 and SK-Hep1 cells through AMPK activation

**Figure 5:**
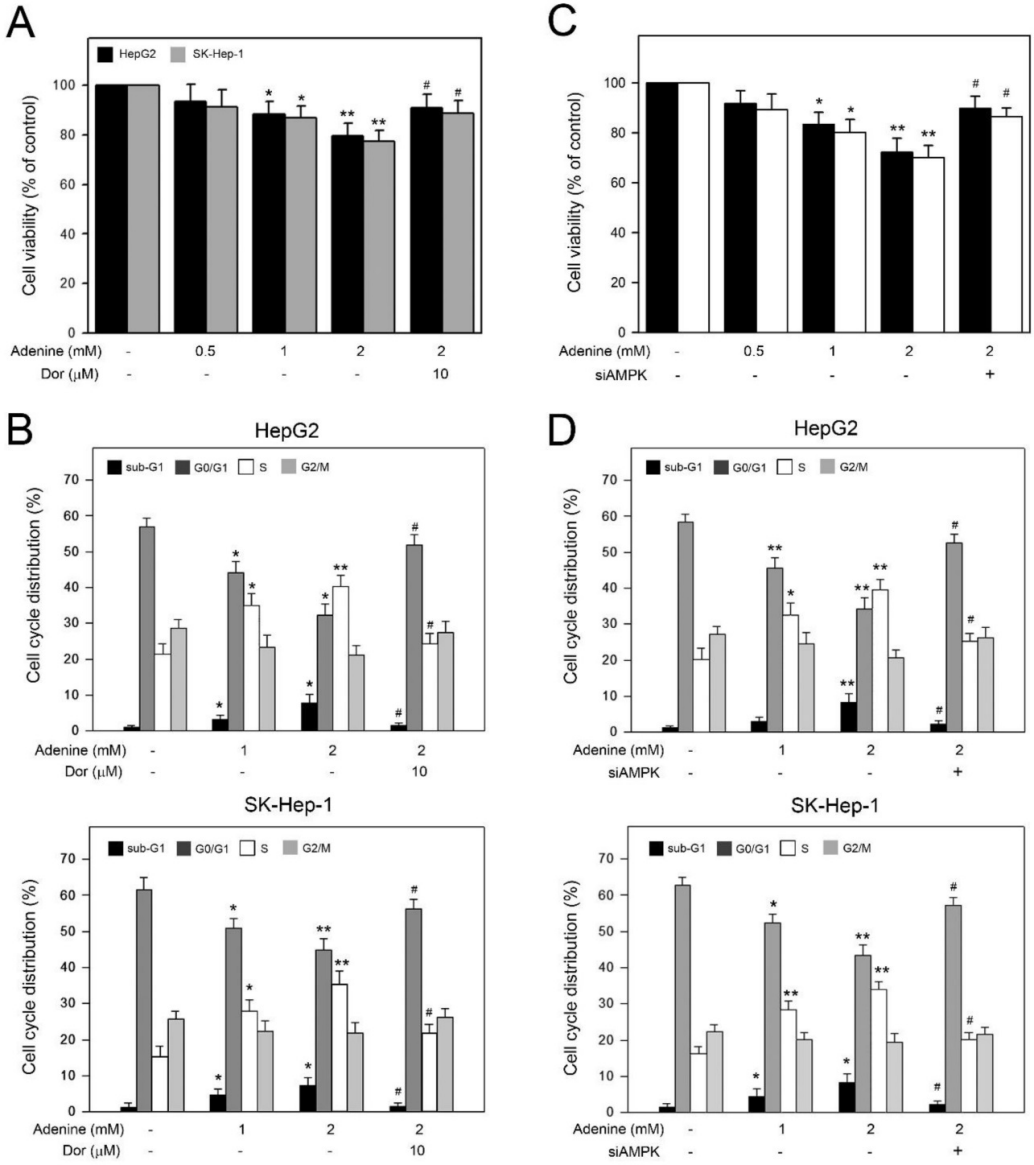
Involvement of AMPK activation in the reduced viability and the cell cycle arrest in HepG2 and SK-Hep-1 cells in response to adenine. Cells were pre-treated with or without dorsomorphin (Dor) for 2 h, treated with adenine at 1 or 2 mM for 24 h, and then collected for (A) cell viability assay, and (B) flow cytometric analysis. Cells were transfected with scramble RNA or siAMPK, treated with adenine at 1 or 2 mM for 24 h, and then collected for (C) cell viability assay, and (D) flow cytometric analysis. Data were expressed as mean ± S.D. from three independent experiments. * and **, *P*<0.05 and *P*<0.01 as compared to PBS control. #, *P*<0.05 as compared to 2 mM adenine alone.

Basing on the observation of adenine inducing AMPK-mediated apoptotic signals in HepG2 cells, we further explored the involvement of AMPK activation in the decreased cell viability and accumulated S phase in HCC cells in the presence of adenine. As shown in Figure 5A, 2 mM adenine treatment clearly reduced the cell viability of HepG2 and SK-Hep-1 cells to 78.5 ± 5.1% and 76.3 ± 4.2%, respectively (*P*<0.01). In addition, by pretreating with dorsomorphin, the adenine-reduced cell viability was restored to 90.7 ± 5.7% and 88.5 ± 4.7%, respectively (*P*<0.05). Similarly, cell cycle distribution analysis showed that 2 mM adenine treatment resulted in increased S phase ratios from 20.8 ± 2.5% to 41.2 ± 2.8% (*P*<0.01, HepG2) and from 16.7 ± 2.2% to 36.2 ± 4.3% (*P*<0.01, SK-Hep-1) (Figure 5B). By pretreating with dorsomorphin, the adenine-increased S phase ratio was lowered to 24. ± 2.6% (*P*<0.05, HepG2) and 21.7 ± 2.1% (*P*<0.01, SK-Hep-1) (Figure 5B). Similarly, we silenced the AMPK expression in HepG2 and SK-Hep-1 cells by using specific siRNA, and the results showed that AMPK knockdown significantly restored the cell viability and S phase accumulation in both HCC cells (Figure 5C and 5D). Taken together, these observations show that AMPK activation is involved in the decreased cell viability and the S phase arrest in HCC cells in response to adenine treatments.

## Discussion

Mounting evidences have shown that dysregulation of metabolic pathway, particular the energy sensing, is highly associated with carcinogenesis in several types of carcinomas such as renal cell carcinoma (RCC) and pancreatic carcinoma 15, 16. AMPK plays a pivotal role in sensing and regulating cellular energy level in all eukaryotic species. For example, under nutrient deprivation and hypoxia conditions, low intracellular ATP level induce AMPK activation and the following signaling cascade to promote cellular energy production 17. In addition to energy regulation, AMPK also governs the important cellular physiological activities including cell proliferation, cell growth, and autophagy 18, 19. Accordingly, AMPK has been recognized as an important anticancer target in suppressing the growth of different carcinoma cells such as 786-O 10, K562 11, and HT29 20. Similarly, we also observe that adenine-induced AMPK activation play an important role in the growth inhibition of HepG2 and SK-Hep-1 cells which express wild-type p53. On the contrary, adenine dose not influence the growth of p53-deficient Hep3B cell. Accordingly, it suggests that AMPK may act as a potential target to promote the treatments for those HCCs with wild-type p53 expression.

The p53 is tumor suppressors that governs cell cycle progression and play an important role in triggering apoptotic signals and the consequent cell apoptosis in response to various stresses 21, 22. Although p53 induces cell cycle arrest at G0/G1 phase in response to extrinsic stresses, p53 activation also contributes to S phase arrest via p21-mediated inhibition of CDK2 23. Consistently, our results also show that adenine induces p53 activation, upregulates p21 expression, and decreases CDK2 expression, which may induce the S phase arrest of HCC cells. Cell apoptosis is a primary anticancer effect of most chemotherapy drugs and many anticancer drugs that can induce p53-mediated apoptosis have been developed 24, 25. In addition, previous studies have reported that genetic manipulation of LKB1, an AMPK upstream activator, is essential for the development of HCC 26 and the AMPK/p53 axis is crucial to the metformin-induced apoptosis of HepG2 cell 27. In this study, we demonstrate that adenine induces AMPK activation and the subsequent p53/p21/PUMA pro-apoptotic signaling, leading to the S phase arrest and inhibited cell growth of both HepG2 and SK-Hep-1 cells. These findings not only suggest that adenine possesses anticancer potential against HCC cells with wild-type p53 but also indicate that AMPK/p53 axis may be a promising target to augment the treatments for HCCs with wild-type p53.

p21 is a cyclin-dependent kinase inhibitor that can be induced by p53 and inhibits cyclin E/CDK2, resulting in G1 phase accumulation. Overexpression of p21 may also inhibit CDK1 and prevent G2/M transition. Moreover, p21 has also been demonstrated to inhibit the kinase activity of cyclin A/CDK2, thereby inhibiting the cell cycle through S phase arrest 28, 29. Therefore, numerus studies have shown that p21 plays a central role in carcinogenesis-associated cell cycle regulation and DNA repair 30. In this study, we observe that inhibition of adenine-induced p53 expression partially reduces the upregulation of p21, suggesting that p53-independent p21 upregulation may also be involved in the adenine-mediated cell cycle inhibition and apoptotic signaling.

In conclusion, our findings provide evidences that adenine can suppress wild-type p53 HCC cell proliferation via AMPK-mediated p53/p21 cascade and the resulting induction of S phases arrest and apoptotic signaling. By augmenting both arms of cell cycle inhibition and apoptotic signaling, adenine exhibits a promisingly antiproliferative potential against human HCC cells.
